# Ozone-Mediated Washing Process of Reference Stain Textile Monitors

**DOI:** 10.3390/polym17141906

**Published:** 2025-07-10

**Authors:** Tanja Pušić, Vanja Šantak, Tihana Dekanić, Mirjana Čurlin

**Affiliations:** 1 University of Zagreb Faculty of Textile Technology, Prilaz baruna Filipovića 28a, 10000 Zagreb, Croatia; tihana.dekanic@ttf.unizg.hr; 2 Ammonia d.o.o., Ulica Vlahe Bukovca 34, 10290 Zaprešić, Croatia; vanja.santak@gmail.com; 3 University of Zagreb Faculty of Food Technology and Biotechnology, Pierottijeva 6, 10000 Zagreb, Croatia; mirjana.curlin@pbf.unizg.hr

**Keywords:** washing process, detergent, ozone, monitors, stain removal, sanitation

## Abstract

The complex chemical composition of certain color stains on textiles requires an optimal proportion of thermal and chemical action in the Sinner cycle of the washing process. In this study, both factors were analyzed by varying the composition of the liquid detergent, bleach, and ozone at temperatures of 30 °C, 40 °C, 60 °C, 75 °C, and 90 °C. Standard cotton fabrics stained with tea, red wine, and blood/milk/ink were selected as monitors, which were evaluated before and after the washing process by spectral parameters. The data sets and their interrelationships were evaluated by a cluster analysis (CA) and ANOVA. An unstained standard cotton fabric was selected as a reference for qualification of the sanitation effect. The stain removal effects showed a selective influence of ozone in the washing processes under the investigated conditions, including the synergy of standard materials—stain monitors and different Sinner cycle factors. The most effective sanitation was achieved in processes using formulations with higher concentrations of liquid detergent (D) and bleaching agents (BA) across all tested temperatures. A lower ozone concentration in combination with lower concentrations of detergents and bleaching agents in washing processes at 30 °C and 40 °C also contributed positively to the effect on sanitation.

## 1. Introduction

The cleaning of textiles stained with unwanted substances (soil) that have accumulated on the surface or in fibrous structure is described by the term detergency or washing performance [1]. The removal of soil or stains is the reverse process of coagulation and adhesion, which requires an impulse to loosen the dirt from the surface [2]. The interaction between the soiled textiles and the operational parameters of the washing process impact on the washing performance and the lifetime of the textiles [3,4]. The amount of water is represented by the inner cycle, which connects four factors of the washing process: mechanical movement, duration, temperature, and chemical action.

Modern washing agents or detergents are aimed at improved multifunctional results, manifested by high levels of cleanliness, maintaining the quality of textiles, and ensuring sustainability. The most important ingredients of modern detergents include surfactants, enzymes, bleaches, and their activators [3,4,5].

Due to their ability to reduce the surface tension of water, surfactants (SAS) enable better contact between the detergent ingredients in the washing bath and the soil on the textile. These molecules consist of a hydrophilic head and a hydrophobic tail, which enable them to emulsify fats and oils and remove them from textiles. Anionic, non-ionic and amphoteric SAS are most commonly used in detergents. Anionic surfactants are negatively charged, effectively remove various stains from textiles, have a high foaming capacity and a strong effect on fats and oils. They are able to chelate calcium and magnesium ions from water, so it is important to use a good builder in detergent formulations. Non-ionic surfactants have no charge and are effective at removing fats and oils stains, have a low tendency to foam and an excellent effect in combination with anionic surfactants [5]. Amphoteric surfactants have good compatibility with non-ionic surfactants and are acceptable for low performance liquid detergent formulations [6,7].

Enzymes as biocatalysts have played a key role in detergents for decades, as they not only accelerate chemical decomposition reactions, but are also involved in the decomposition of complex molecules into smaller and soluble molecules that can be easily rinsed away. The specificity of an individual enzyme is shown by the fact that it only acts on certain types of molecules, i.e., it only accelerates a certain reaction. The action of enzymes according to the principle that the key (the molecule) enters the lock (the active site of the enzyme) is largely dependent on pH and temperature. Different stabilization techniques enable a controlled release of the enzyme and protection against denaturation during storage [3,5,7,8,9].

Bleaching agents and their activators are important components of powder detergents due to their ability to remove colored stains adhering to textiles by oxidative or reductive decomposition of chromophore systems. Optimal concentrations of sodium percarbonate and hydrogen peroxide considered as environmentally friendly ingredients as they break down into oxygen and water without producing harmful by-products [10].

The conjugated chains in the molecule linked with the color, can also be broken down by action of ozone, which is compatible with bleaching agents and contributes to the whiteness of washed textiles [11]. The unstable ozone molecule (O_3_) decomposes rapidly into oxygen, especially in the presence of heat or other catalysts [12,13,14,15,16]. The released oxygen atom produces a reactive oxygen radical that can oxidize other substances, viruses, bacteria, fungi, organic molecules (olefin double bonds, acetylene triple bonds, aromatic compounds, phenols, various types of dyes with polycyclic aromatic and complex structure) and inorganic ions [17,18].

The development of detergent formulations focuses on improving efficiency, safety, and sustainability through the introduction of biodegradable ingredients (SAS, enzymes), active ingredients for low temperatures (bleach activators, enzymes) and hypoallergenic ingredients. Sustainability is reflected in the energy and environmental benefits resulting from (i) the ability to wash and sanitize at lower temperatures, (ii) decomposition into oxygen leaving no harmful residues in the environment, and (iii) extension of durability properties of textiles. The use of ozone in the washing process contributes to disinfection and provides some savings by reducing washing time by 20% and chemicals by 45% [19]. Despite the emphasized advantages, its use in the washing process is associated with some challenges, especially in terms of stability and safety. Important process parameters in the production of ozone are its concentration and yield as well as energy efficiency [20]. Its lifetime is several hours in air and half an hour in water at room temperature [19]. Due to low stability, it must be generated on site, which requires specialized equipment. High concentrations of ozone can be harmful to health, so adequate ventilation must be ensured.

In accordance with the mentioned properties, the use of ozone in the washing process can contribute to the stain removal while improving the overall sustainability of the process [21].

The quality assessment of the washing performance can be based on (i) stain removal, (ii) whiteness retention, and (iii) textile protection (color care, dye transfer, fabric care, damage, tear resistance, burst resistance, deposits, etc.) [22].

This study highlights the combined chemical potential of liquid detergent, bleaching agent, and ozone, each applied at two concentration levels, in enhancing stain removal and sanitation efficiency during the washing of standard cotton-stained monitors across various temperatures.

## 2. Materials and Methods

### 2.1. Material

Three different test stains (tea, coffee, and an aqueous soil extract) are required for the evaluation of bleaching agents in the washing process [23]. For this purpose, three standard soiled fabrics—cotton swatches (WFK 10 J tea, WFK 90 LI red wine, EMPA 116 sterilized blood/milk/ink) were used as monitors for the study.

A description of the EMPA (German Eidgenössische Materialprüfungs und Forschungsanstalt, St. Gallen, Switzerland), and wfk—Testgewebe GmbH (German Wissenschaftliches Forschungsinstitut für Reinigungstechnologie, Brüggen-Bracht, Germany), standard cotton monitors [24,25] is given in Table 1.

Bleached reference cotton fabric (100% CO), WFK 11 A in tabby weave, mass per unit area of 180 g/m^2^, density of 27 yarns in warp and weft direction, both textured yarns with a count of 29.5 tex before and after washing under different conditions was used for the evaluation of sanitation.

Washing process

The selected monitors were washed with 6 kg of cotton ballast, varying the time, temperature, concentrations of liquid detergent, bleaching agent, and ozone.

The washing process was carried out in a washing machine, Giant-C MAX, LG, South Korea, equipped with an ozone generator, BWO^3^, dosed in two concentrations, the first being 0.5 to 1.0 mg/L and the second 0.5 to 2.0 mg/L in the water for the prewash and the final water for rinsing at temperatures of up to 20 °C.

The agents used in the washing process were liquid detergent (D) and bleaching agent (BA), both from the Belgian company Christeyns.

The composition of the detergent according to the EC Directive contains anionic and amphoteric surfactants (<5%), soap and non-ionic surfactants (5–15%), polymers, optical brighteners, and fragrances.

As the removal of specific color stains by liquid detergents is incomplete, it requires the use of a bleaching agent as an additive (BA) formulated in accordance with the EC Directive, containing non-ionic surfactants (<5%), sodium percarbonate (>30%), complexing agents, and fragrances.

The dosing of liquid detergent (D), bleaching agent (BA), and ozone (O_3_) as well as the labels of specific washing processes, are specified in Table 2 while the duration and temperature is given in Table 3.

### 2.2. Methods

The effects of detergent concentration, bleaching agent, ozone, and temperature in the washing process on stain removal was assessed by remission spectrophotometry and the sanitation by analyzing the presence of aerobic bacteria according to the standard protocol for water samples [26].

Stain removal

Stain removal was monitored by analyzing the spectral properties of standard monitors before and after washing using a Spectraflash SF300 remission spectrophotometer, DataColor, Rotkreutz, Switzerland, with a standard light source D_65_ imitating daylight and a geometry d/8° (diffuse illumination at an observation angle of 8°). For the stain removal analysis, the reflectance value at a wavelength of 460 nm (R_460_) was measured at four randomly selected locations, with the higher value indicating better washing performance on the standard monitor. The results are presented as an average of four individual measurements with error bars for all wash temperatures [27,28]. An ANOVA analysis of variance was also performed in MS Excel software to determine if there was a statistically significant difference between the temperatures for each wash process.

Cluster analysis

The data sets and their correlations were analyzed using a cluster analysis (CA), a statistical method of dividing a group of objects into classes so that similar objects are in the same class. A cluster analysis searches for objects that are close to each other in the variable space [29,30]. This method is characterized by an unknown number of clusters, which are determined on the basis of a dendogram, a graphical representation of the gradual grouping of objects into clusters, on which the distances between the individual levels can be observed. All analysis were performed in TIBCO Statistica^®^ Software.

Sanitation effect

The sanitation effect was shown by the results of microbiological analysis of the reference cotton samples (WFK 11A) before and after the washing process (A, A-O, B, B-O, A-OO, B-OO) and the variation in temperature (30 °C, 40 °C, 60 °C, 75 °C, 90 °C).

The analysis was carried out by modified method [31]. The most important nutrients were tryptone and yeast extract (without glucose), which were used for the enumeration of microorganisms in the water, as they better reflect the natural nutrient conditions in the water.

Microbiological analysis was performed by inoculating unwashed (U) and washed samples onto prepared yeast extract agar plates. After 44 h of incubation at 37 °C, the plates were analyzed by visual inspection and colony counting. The method for the sanitation effect was performed as a modified method of microbiological water analysis. Instead of 1 mL of solution as prescribed by the standard, in this case 1 cm^2^ of the tested fabric sample was taken, which is why the results were not expressed as CFU/mL, but were objectively evaluated as a relative ratio of number of colonies (all samples) and expressed descriptively as sanitation effect, as shown in Table 4.

## 3. Results and Discussion

### 3.1. Stain Removal

**Figure 1:**
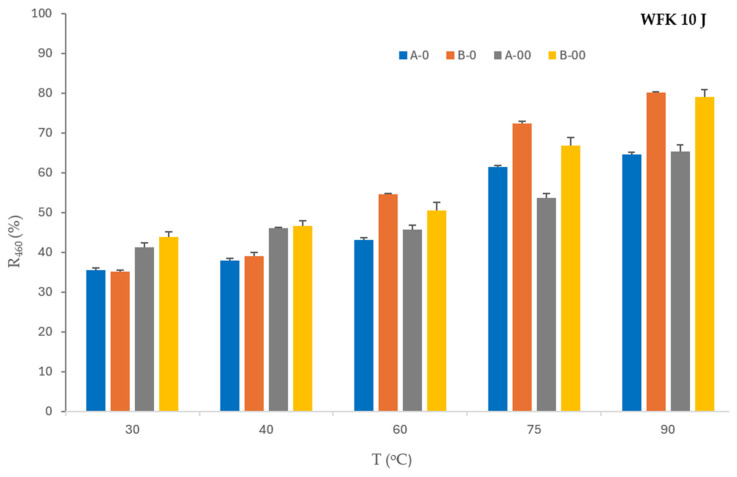
Reflectance of the WFK 10 J monitor washed in ozone-mediated processes.

**Figure 2:**
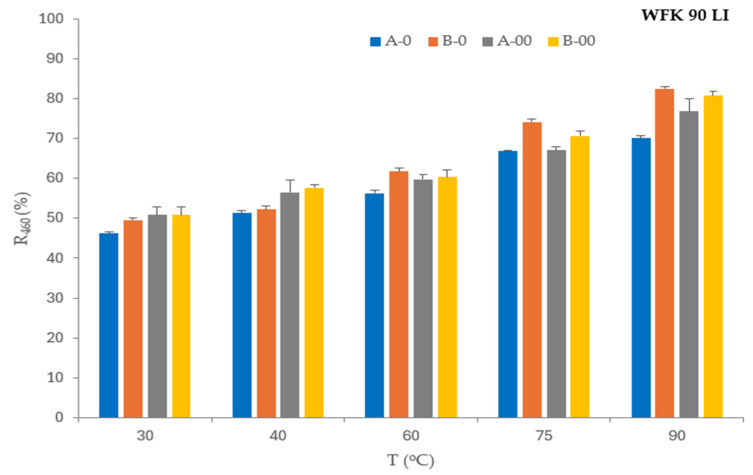
Reflectance of the WFK 90 LI monitor washed in ozone-mediated processes.

**Figure 3:**
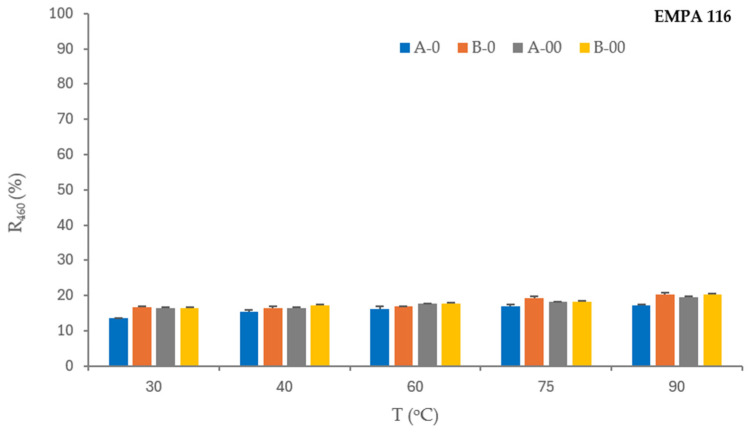
Reflectance of the EMPA 116 monitor washed in ozone-mediated processes.

The influence of detergent and bleaching agent concentration in formulations (A, B) and their ozone-mediated effect (A-O, A-OO, B-O, B-OO) on stain removal from standard monitors—cotton fabrics colored with tea (WFK 10 J), red wine (WFK 90 LI), and blood/milk/ink (EMPA 116) at temperatures of 30 °C, 40 °C, 60 °C, 75 °C, and 90 °C is shown in Figure 1, Figure 2 and Figure 3.

WFK 10 J is a reference monitor—cotton fabric stained with tea of unknown composition. According to [32], many bioactive compounds such as polyphenols, pigments, polysaccharides, alkaloids, free amino acids, and saponins have been identified in tea, where the quantity of these ingredients depend on the tea category.

Stains (e.g., a natural dye from tea, wine, or fruit juice) from the surface are broken in a redox process enabled by a bleaching agent [32,33].

The reflectance values of WFK 10 J before (U) and after washing (A, B, A-O, A-OO, B, B-O, B-OO) in combination with the temperatures are shown in Table 5.

The reflectance values show that a higher concentration of detergent and bleaching agent (B) improves the removal of tea from a monitor at all temperatures compared to lower concentrations of detergent and bleaching agent (A). The washing process with a lower concentration of detergent and bleach, supported by a lower concentration of ozone (A-O), enhances the removal from the analyzed monitor only at 75 °C. The washing process with a high concentration of detergent and a lower concentration of ozone (B-O) does not improve the performance compared to the washing process with a high concentration of detergent and bleach without ozone (B). The effect of a higher concentration of ozone (A-OO, B-OO) improves the removal of soiling from tea at 30 °C and 40 °C compared to A-O and B-O.

Figure 2 shows results of the influence of ozone on the reflectance values of the washed WFK 10 J monitor in dependence of temperatures.

Figure 1 shows the influence of lower (O) and higher concentrations of ozone (OO) with lower (A) and higher concentrations of detergent and bleaching agent (B). The reflectance values of WFK 10 J of the washed monitor confirm that both concentrations of ozone are an equally good mediator at both concentrations of detergent and bleach at lower temperatures (30 °C and 40 °C). Accordingly, it was not necessary to increase the dosage of D and BA for this type of stain monitor. The figure clearly shows that the increased concentration of ozone (OO) with lower and higher concentrations of detergent and bleaching agent (A, B) only increases the reflectance values at temperatures of 30 °C and 40 °C, i.e., favors the removal of the tea stain from the cotton monitor. Lower concentrations of detergent, bleach, and ozone (A-O) improve the tea stain removal effect only at 75 °C, while a higher concentration of detergent and bleach with a lower concentration of ozone (B-O) increases the reflectance values in the washing process at 60 °C, 75 °C, and 90 °C.

WFK 90 LI is a standard cotton monitor stained with red wine. The compounds isolated from red wine are pyranoanthocyanins, where the pyran ring stabilizes the structure and provides a more intense color than anthocyanin dyes [33].

The primary effect in the washing process of cotton fabric stained with red wine is improved by increasing the temperature in almost all analyzed washing process conditions (A, B, A-O, B-O, A-OO, B-OO). A small decrease in the reflectance value was recorded only for WFK 90 LI fabric washed with a smaller concentration of detergent and bleach at 90 °C (A). The influence of a smaller ozone concentration (O) with a high concentration of agents in washing bath B is insignificant. The influence of a higher ozone concentration (OO) with a higher concentration of detergent and bleaching agent compared to B and B-O is favorable only at 30 °C and 40 °C. Lower concentrations of agents (A) supported by a higher concentration of ozone (OO) improves the reflectance values of washed WFK LI fabric at all analyzed temperatures, Table 6. The washing process with a higher concentration of detergent and bleach with a lower concentration of ozone (B-O) compared to B does not favor the effect of removing wine stains at the analyzed temperatures, while a higher concentration of ozone (B-OO) favors the effect at 30 °C and 40 °C.

Figure 2 shows more clearly that a higher concentration of ozone (OO) with a less concentration of detergent and bleaching agent (A) favors the removal of wine stains only at 30 °C, while a higher concentration of detergent and bleach (B) increases the reflectance values, i.e., favors the removal of wine stains from cotton monitors at temperatures of 30 °C and 40 °C.

The statement already made in the evaluation of the WFK 10J stain monitor that it is not necessary to increase the dose of D and BA for the WFK 90 LI does not apply. This confirms that it is necessary to monitor the influence of ozone in the washing process in combination with a specific stain monitor and it is difficult to draw general conclusions. The best effect is achieved with the B-O washing process at 90 °C. When observing the influence of temperature on the stain removal performance in the analyzed washing processes, it can be seen that the optimal combination depends on temperature, so the A-OO at 30 °C, the B-OO at 40 °C, the B-O at 60 °C, the B-O at 75 °C, and the B-O at 90 °C. As a summary, the best combination for removing wine stains at 60 °C, 75 °C, and 90 °C is a higher concentration of detergent and bleaching agent with a less concentration of ozone (B-O). A higher concentration of ozone favors the removal of wine stains at lower temperatures, 30 °C (A-OO) and 40 °C (B-OO).

Less concentrations of detergent, bleaching agent, and ozone (A-O) improve the tea stain removal effect only at 75 °C, while a high concentration of detergent and bleaching agent with a less concentration of ozone (B-O) increases the reflectance values in the washing process at 60 °C, 75 °C, and 90 °C.

EMPA 116 is a monitor stained with heterogenic mixture of blood/milk/ink for testing detergency and bleach effect in a washing process. The removal performance of blood/milk/ink stains from cotton fabric—reflectance values of EMPA 116 before and after the washing processes as a function of the observed variables is shown in Table 7. The washing process of cotton fabric stained with a blood/milk/ink mixture (EMPA 116) requires a synergy of components that ensure the detergency and bleaching effect. Blood is a stain that requires a decolorizing agent, although its removal can sometimes be problematic [5]. Stubborn protein stains on textiles from sources such as milk, cocoa, blood, egg, egg yolk, and grass are difficult to remove with non-enzymatic detergents, especially when the stains have dried and aged. Ink is a solution or suspension of suitable dyes or pigments [34] that must be decolorized during the washing process.

The stain removal from the standard monitor EMPA 116 (cotton fabric soiled with a mixture of blood, milk, and ink) is extremely poor under all washing process conditions. From the reflectance values in Table 7, it is evident that the bleaching agent in both concentrations was not efficient. It means that the protein component (blood/milk) predominates over the ink as a colorant, which requires proteolytic enzyme activity in the washing process that liquid detergents do not contain. The results obtained are consistent with earlier research findings, where it is proved that all tested standard soils, with the exception of EMPA 116, were washed better with hydrogen peroxide [10].

The one-way ANOVA focusses on the evaluation effects of temperature for each washing process. Results of the ANOVA analysis, Table 8, show that there is a statistically significant difference in relation to the temperature during the washing process, *p* value < 0.05, and F value > F crit.

The analysis of the sum of reflectance values (ΣR_460_) of all three washed monitors (WFK 90 LI, WFK 10 J and EMPA 116) is shown in Table 9.

A higher ozone concentration combined with less concentrations of detergent and bleaching agent (A-OO) favors the achievement of the overall effect in the washing process at temperatures of 30 °C, 40 °C, 60 °C, and 90 °C. A higher ozone concentration in combination with a higher concentration of detergent and bleach (B-OO) is suitable at 30 °C and 40 °C. The washing process conducted with a higher concentration of detergent and bleach mediated by less ozone concentration (B-O) at 60 °C and 90 °C is the best combination.

**Figure 4:**
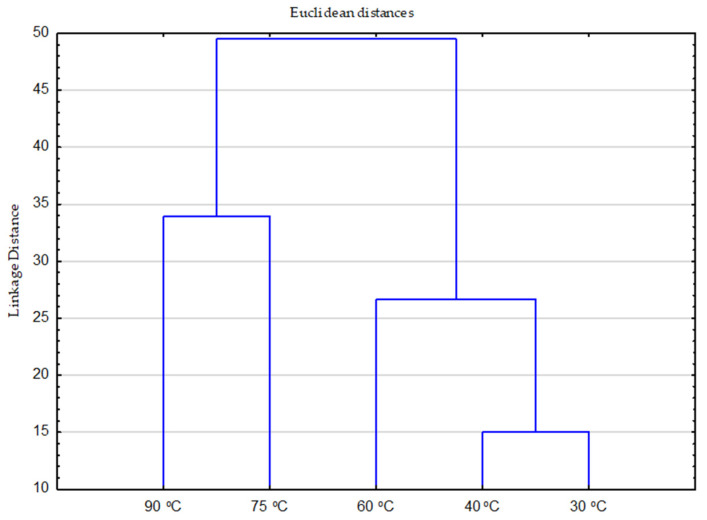
The distribution by group for temperature as a variable of formulations, ozone mediated formulations and all stain monitors.

**Figure 5:**
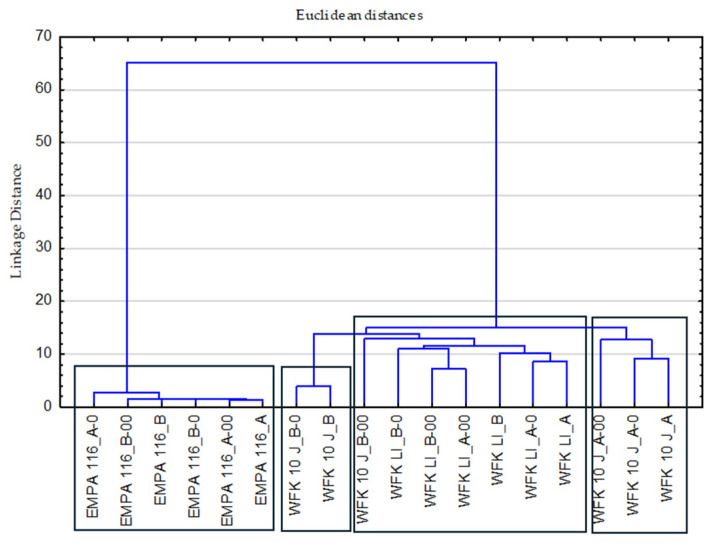
The distribution by group to show all stains and all detergent variables covers all temperatures.

Analysis of the combined effect of all chemistry and temperature parameters on individual stains, Figure 4 and Figure 5.

The effect of temperature is illustrated by the expected standard distribution from lower to higher temperatures, Figure 4.

The influence of chemistry (A, B, A-O, B-O, A-OO, B-OO) on a single stain in Figure 5 is characterized by a different grouping. For the reference-stained monitor, EMPA 116, small differences in the effects were observed. Furthermore, the influence of chemistry on the WFK 10 J reference monitor is visible in the different distribution and grouping for low and high concentrations of detergent with bleaching agent and for low and high concentrations of ozone. Homogeneous grouping is characteristic for stain reference WFK 90 LI analyzed through the impact of chemistry.

### 3.2. Sanitation Effect

**Figure 6:**
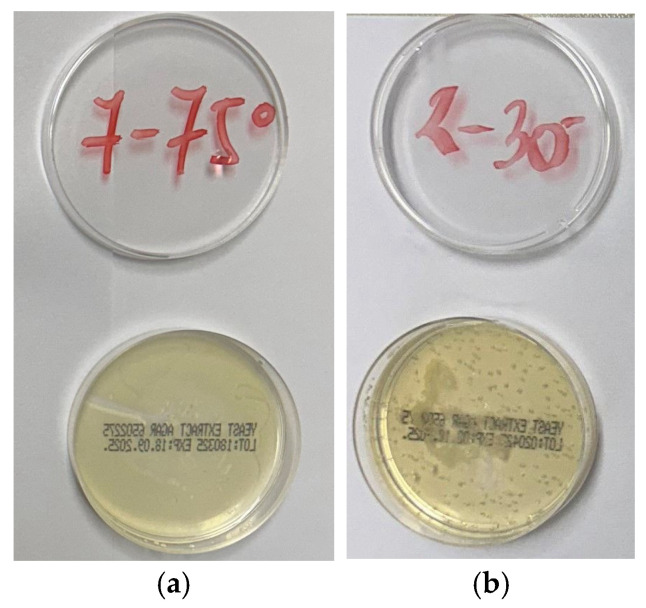
Sanitation effect (**a**) very low pollution and score 5; (**b**) high pollution and score 2.

The classification of contamination on standard cotton fabric was based on bacterial accumulations on a nutrient Agar medium after 44 h of incubation at 37 °C. Images with high and very low levels of pollution are shown as examples of the effects of sanitation in Figure 6, for which ratings and a linguistic description are given in Table 10.

The sanitation effect achieved by higher concentrations of liquid detergent (D) and bleaching agent (BA) predominated at all temperatures in the washing processes (B) and no improvement was observed by the mediation of both ozone levels at the temperatures investigated (B-O, B-OO). A favorable effect of ozone on the sanitation effect was achieved in the processes with lower doses of detergent and bleaching agent (A) at 30 °C and 40 °C.

## 4. Conclusions

Investigations of the stain removal effect and the sanitation effect in the washing process by varying the detergent, bleach, and ozone concentrations at temperatures of 30 °C, 40 °C, 60 °C, 75 °C, and 90 °C revealed a selectivity that is achieved through the interaction of standard monitors—soil carriers and Sinner’s circle factors.

The performance of all washing processes of the reference stain EMPA 116 (cotton fabric soiled with a mixture of blood, milk, and ink) at all temperatures is extremely weak despite the presence of a bleaching agent.

The formulation of detergent and bleach in higher concentrations (B) improved the removal of tea stains at temperatures of 60 °C, 75 °C, and 90 °C. The mediation effect of ozone was not observed compared to bleach and detergent. The mediation of a lower ozone concentration was found at lower concentration of detergent and bleach (A-O) only at temperatures of 30 °C and 40 °C, while a higher ozone concentration (A-OO) increased the cleaning effect at 75 °C for one class.

Despite the different effects of the parameters, the influence of temperature proved to be statistically significant.

The best sanitation effect was achieved at all temperatures tested in processes with a higher concentration of detergent and bleaching agents (B), and no improvement was achieved by the mediation of ozone.

The research results indicated the need for analyses and other agents in the washing process, primarily enriched with modern multi-enzyme complexes in combination with ozone. In addition, the sanitation effect indicates the need for further research to quantify the degree of reduction in individual Gramme-positive and Gramme-negative bacteria.

The ozone-mediated washing process was effective in the stain removal at lower temperatures, making it is possible to save energy. However, as the production of ozone is an energy-intensive process, this will also be taken into account in future research.

## Figures and Tables

**Table 1:** Designation and specification of soiled standard cotton monitors.

Type of Soiled Standard Cotton Monitors			Effect
WFK 10 J	tea	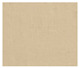	Bleaching
WFK 90 LI	red wine	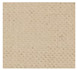	Bleaching
EMPA 116	sterilized blood/milk/ink	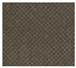	Detergency/Bleaching

**Table 2:** Labels and conditions of the washing process with lower and higher concentrations of detergent, bleach, and ozone.

Washing Process	Dosage		
	D (mL/kg)	BA (g/kg)	O_3_ (mg/L)
A	8.3	6.7	-
A-O	8.3	6.7	≤1
A-OO	8.3	6.7	≤2
B	15.0	10.0	-
B-O	15.0	10.0	≤1
B-OO	15.0	10.0	≤2

**Table 3:** Temperature and time of all washing processes.

T (°C)	30	40	60	75	90
t (min)	51	61	69	84	99

**Table 4:** Classification of contamination and evaluation (1–5) of the cleanliness of the samples (sanitation effect), 1—unclean; 5—very clean.

Level of Contamination	Grade
Very low	5
Low	4
Medium	3
High	2
Very high	1

**Table 5:** WFK 10 J—tea stain removal performance of cotton monitor before (U) and after washing processes (A, B, A-O, B-O, A-OO, B-OO).

T (°C)	A	A-O	A-OO	B	B-O	B-OO
30	31.6	35.51	39.01	33.85	35.19	41.44
40	35.67	38.06	45.72	39.42	39.08	44.36
60	42.03	43.15	43.23	51.77	54.63	47.56
75	53.79	61.52	51.70	71.16	71.53	63.93
90	63.16	64.69	62.16	82.53	80.24	75.98
U	35.93	35.93	35.93	35.93	35.93	35.93

**Table 6:** WFK 90 LI—red wine stain removal performance of cotton monitor before (U) and after washing processes (A, B, A-O, B-O, A-OO, B-OO).

T (°C)	A	A-O	A-OO	B	B-O	B-OO
30	45.37	32.45	56.89	49.07	49.53	54.35
40	48.82	46.21	51.33	53.34	52.28	56.39
60	53.54	51.33	57.75	60.48	61.69	57.46
75	70.99	56.23	65.61	74.99	74.09	68.32
90	66.31	66.8	72.78	84.38	82.45	78.96
U	32.45	32.45	32.45	32.45	32.45	32.45

**Table 7:** EMPA 116—blood, milk, and India ink stain removal performance of cotton monitor before (U) and after washing processes (A, B, A-O, B-O, A-OO, B-OO).

T (°C)	A	A-O	A-OO	B	B-O	B-OO
30	16.1	15.41	15.73	15.57	16.66	16.57
40	15.87	16.29	16.46	16.92	16.53	17.16
60	16.77	16.91	17.65	17.21	16.89	17.65
75	18.91	17.32	18.11	19.76	19.22	17.53
90	19.14	16.81	19.36	18.91	20.32	20.28
U	13.51	13.51	13.51	13.51	13.51	13.51

**Table 8:** ANOVA analysis of variance.

Washing Process	ANOVA		
	F	*p*-Value	F crit.
A-O	3.065	0.015	2.354
A-OO	2.515	0.028	2.219
B-O	4.791	0.008	2.354
B-OO	3.516	0.004	2.219

**Table 9:** Reflectance values of all washed monitors—the overall effect.

ΣR_460_ (WFK 90 LI, WFK 10 J and EMPA 116)					
	30 °C	40 °C	60 °C	75 °C	90 °C
A	93.07	100.36	112.34	143.69	148.61
A-O	97.13	105.68	116.39	145.64	154.30
A-OO	111.63	113.51	118.63	135.42	163.97
B	98.49	109.68	129.46	165.91	171.77
B-O	101.38	107.89	133.21	164.84	184.94
B-OO	112.36	117.91	122.67	149.78	175.22

**Table 10:** Sanitation effect on the washing processes.

Sample	30 °C	40 °C	60 °C	75 °C	90 °C
U	1				
A	2	1	3	3	3
B	5	5	5	4	5
A-O	3	3	3	2	3
B-O	4	3	5	4	4
A-OO	2	3	3	3	3
B-OO	2	3	3	3	4

## Data Availability

The original contributions presented in this study are included in the article. Further inquiries can be directed to the corresponding author.
